# Visibility and Well-Being in School Environments: Children’s Reflections on the “New Normal” of Teaching and Learning during the Covid-19 Pandemic

**DOI:** 10.1007/s42448-022-00136-7

**Published:** 2023-01-12

**Authors:** Susann Fegter, Miriam Kost

**Affiliations:** Institute of Educational Science, Technische Universität Berlin, Sekr. FH 5-1, Fraunhofer Straße 33-36, 10587 Berlin, Germany

**Keywords:** Child well-being, Children’s perspectives, School well-being, Visibility, Covid-19 pandemic, Digitization, Distance learning

## Abstract

This paper aims to contribute to the theory on school-related well-being by applying a qualitative approach that focuses on children’s experiences during the Covid-19 pandemic and conceptualizes them as an epistemic opportunity to reconstruct aspects of school-related well-being from children’s perspectives. Within the framework of the multinational qualitative study Children’s Understandings of Well-being (CUWB), it conceptualizes well-being as a cultural construct and argues for including children’s voices in the process of knowledge production. By drawing on statements from online interviews with 11- to 14-year-old children from Berlin, Germany in spring 2021 during school lockdown and by using a discourse analytical approach, the paper outlines the findings on visibility as a central feature of well-being in school environments that children make relevant for experiences of agency, security, and self. Visibility in school is constructed as a medium of control that subjects their bodies to norms of the school, exposes the individual to the gaze of others, and provides security in the context of the digital sphere and its temptations. The paper argues to systematically include these reflections and assessments of new digital learning arrangements during the Covid-19 pandemic into theoretical concepts on school-related well-being.

## Introduction 

When schools in Germany closed from one day to the next in March 2020 as a result of the Covid-19 pandemic, children faced a situation that had never happened before in Germany (Andresen et al., 2020; Budde et al., 2022a, 2022b; Fickermann & Edelstein, 2020). From one day to the next, pupils had to get used to doing school lessons from home. How exactly these lessons looked like and took place was at first largely up to the schools and teachers themselves: some assigned tasks by email, some by phone, and some streamed from the school building to the students’ homes. Only over time did the new arrangements become more regulated, videos and learning software were made available, and rotating teaching models became possible, which were, again, employed very differently by the schools. Despite all the differences, there was one thing in common: familiar routines of schools and school-related learning practices could no longer be applied. All of a sudden, schooling took place in a way completely different than before, and many things that seemed unthinkable before suddenly became possible and a new normality.

This experience of contingency is the starting point of this paper to reconstruct children’s understanding of well-being in school environments from their own perspective. Based on qualitative interviews with 11- to 14-year-old children in Berlin, Germany during pandemic-related school lockdowns, it presents findings from an explorative analysis that focuses, on the one hand, on children’s subjective well-being associated with the school lockdowns and distance learning phases during the pandemic (e.g., what they appreciated and felt good with under the changed learning conditions and what they disliked and not felt good with). It reconstructs secondly the cultural concepts of school-related well-being that are reproduced and addressed in the way how the children talk about their experiences (e.g., structural elements of learning environments they make relevant for their well-being and structural elements of well-being relevant to learning from their perspective).

The paper thus pursues three aims. First, it aims to give children a voice during the pandemic. As Budde and Lengyel (2022) and Andresen et al. (2020) point out, children, during the pandemic in Germany, were not heard very much for a long time and had hardly any representatives who introduced their concerns and experiences into the public discourse. Only recently the first scientific studies have been published that ask about the views of children in Germany during the pandemic (Andresen et al., 2020; Budde et al., 2022b; Hüpping et al., 2022; Langmeyer et al., 2020). This paper aims to contribute to this development by applying a qualitative approach that focuses on children’s experiences with new arrangements of learning and teaching during the Covid-19 pandemic. Second, the paper aims to bring into focus children’s discoveries and insights that they associate with experiences of new normalities during the pandemic. Most studies currently point to the negative impacts of the pandemic on children, such as learning deficits, mental stress, and what children have missed during the school lockdowns (e.g., Bremm & Racherbäumer, 2020; Bujard et al., 2021; UNESCO, 2020), which undoubtedly result from the pandemic. However, there were also good experiences with the “new normality” that children raise that provide indications of positive qualities that children value and appreciate in their everyday lives and in learning environments. With regard to family life and the value of spending time together with the family, these positive impacts have already been elaborated by some of the current studies (e.g., Andresen et al., 2020: 11). With regard to schools, the main focus so far is on negative impacts, and only few studies ask what children have valued and appreciated under the new conditions of learning and teaching (e.g., Wacker et al., 2020). This paper aims to contribute to this perspective by reconstructing advantages that children ascribe to the new normalities and by using these to inform more broadly the theoretical discourse on factors of school-related well-being or well-being in learning environments from the perspective of children.

Third, the paper argues that children’s experiences of contingency during the pandemic with respect to learning and teaching arrangements provide a special epistemic opportunity to develop a better understanding of concepts and factors of school-related well-being from children’s perspective. Our argument is that the experience of contingency during the pandemic, that the established structures and practices of school are possible but at the same time not necessary, as they could also be different, prompt reflections on aspects of school-related well-being that seemed so self-evident before that they were not even mentioned, or that seemed so unchangeable that they were not problematized and made a topic (even if they had an impact on children’s well-being in schools). School in particular represents a manifest institution, strongly embedded in history, society, and former generations of parents and siblings, that it confronts children as a nearly self-evident practice. The pandemic, in contrast, provided experiences for pupils in Germany that were very different, hard to imagine, and nearly unthinkable before the pandemic, for example, that schooling could stop from one day to the other, that school lessons could be done from home for everyone on a regular basis, and that schooling could take place fully digitally or as a hybrid space. Using these experiences of contingency during the pandemic as an epistemic opportunity to inform the theoretical discourse on well-being in learning environments from the perspective of children is the overall aim of this paper. We call this a “methodology from the margins,” as the analytical process starts from the experience of new normalities that were at the margin of what was thinkable or speakable just recently.

The paper is structured in five sections. After the introduction, the “Child Well-Being in Educational Research” section introduces well-being as a category in educational research by distinguishing two different approaches on well-being related to schools and education and by highlighting research gaps on children’s own conceptualizations of well-being in school and learning environments. The “Methodology” section presents the empirical study underlying this paper, outlining the research context and data collection process as well as the methodological approach that refers to the multinational Children’s Understandings of Well-being (CUWB) study (Fattore et al., 2019) in combination with a discourse analytical perspective. The “Findings on Visibility as a Key Element of School-Related Well-Being from Children’s Perspective” section presents findings on the aspect of visibility in learning environments as a relevant aspect of school-related well-being from children’s perspective that are discussed in the last section in the context of the child well-being theory.

## Child Well-Being in Educational Research

Child well-being is a vague or fuzzy concept that has gained importance in recent decades, initially in the field of child indicator research, welfare, and health studies, and addresses the quality of children’s lives, their subjective and objective well-being, and its social, economic, and political preconditions (Ben-Arieh et al., 2014; Fattore et al., 2019). In educational research, the concept of well-being has only recently begun to receive increasing attention. In the following, we suggest to differentiate between two approaches toward child well-being in educational school-related research.

One approach examines well-being as a means or source of successful learning and academic achievement. This approach is supported by international studies that show positive effects of subjective well-being on self-confidence, curiosity, and openness to new situations (Cefai & Spiteri 2021;  Erdem & Kaya 2021; Lavis & Robson 2015; García Bacete et al. 2014; Andresen, 2010) and thus on important preconditions of learning processes. Since 2015, the comparative PISA studies have included student’s well-being in their surveys. PISA 2018 demonstrates, for example, that students who were frequently bullied were more likely to have skipped school and scored lower in reading (Schleicher, 2020). PISA studies also show correlations between subjective well-being and social background. Disadvantaged students and first-generation immigrant students were less likely to report feeling a sense of belonging at school (OECD 2019; for Germany see also Ecarius, 2018). At the same time, there is evidence that a positive school climate can weaken the strong link between socioeconomic status and school success (Berkowitz et al., 2017; OECD 2019). One of the characteristics is that it is often the absence of well-being that comes into focus as a risk factor for learning outcomes, such as fear and bullying. The “move from negative to positive” well-being indicators that has described the broader Child Indicators Movement (Ben-Arieh, 2008) is therefore less of a characteristic for the area of empirical educational research, particularly in the area of international studies measuring academic outcomes and competencies.

The second approach sees well-being as a goal (ends) of education and educational systems. This approach is often found programmatically in school education policies or programs that aim, e.g., “to support pupils’ growth into good and well-balanced people and members of society and to give them the knowledge and skills needed in life” (Konu & Rimpelä, 2002: 80). Examples are the Australian Student Well-being Framework (ASWF) that promotes a vision of Australian schools as “learning communities that promote students’ well-being, safety, and positive relationships so that students can reach their full potential” (Australian Student Well-being Framework, 2018: 1) or the campaign “Improving well-being at school” as part of the Democratic School Network of the European Council (Council of Europe, n.d.). Well-being is defined there as the experience of health and happiness. It includes mental and physical health, physical and emotional safety, and a feeling of belonging, sense of purpose, achievement, and success. This understanding of well-being as a goal of education comes close to the German concept of *Bildung*, which in a humanistic philosophical tradition goes beyond academic achievement or measurable competencies and rather aims to enable self-determined and responsible actions as a human being and a citizen (see Horlacher, 2015).

What both approaches have in common is that they start with a theoretically predefined concept of well-being, which, depending on the background, is located more in psychological or sociological theories and can include subjective as well as objective factors. So far, few studies have asked what children themselves understand by well-being in school environments (and how this relates to social and cultural contexts). Thus, children’s perspectives have hardly been included in conceptualizing and theorizing well-being in school/learning environments. Pioneers are for example Newland et al. (2019) who conducted qualitative in-depth interviews with US children about their perceptions of school climate and school-based interpersonal relationships and the extent to which the children perceived these relationships as supporting or undermining their emotional well-being. The importance of relationships as a central factor for subjective well-being at school from children’s perspective is also investigated by Thomas et al. (2016), who conducted group discussions with students in Australia using Axel Honnet’s theory of recognition. Drake et al. (2021) found out, with data from the Australian CUWB study with 12- to 14-year-old children, that school life has an ambivalent impact on children’s well-being. On the one hand, children note that the opportunities education provides them to pursue self-determined objectives are associated with positive well-being. On the other hand, children discussed how schooling puts pressure on them to meet adult-imposed aspirations and how this undermines their well-being. In the German context and using a quantitative approach, Holzer et al. (2021) conducted a survey among secondary school students and teachers in which the participants provided responses to open-ended questions addressing their general understanding of high and low levels of school-related well-being. Their findings suggest that children’s conceptualizations of school-related well-being include both hedonic (experiences of pleasure and enjoyment) and eudemonic aspects (experiences of meaning and purpose).

The following paper aims to contribute to this new area of research that investigates school-related well-being from children’s own perspective by using qualitative data on children’s perceptions and experiences with school arrangements during the Covid-19 pandemic. The idea, as outlined above, is that the experience of new routines and very different school practices has an epistemic potential for learning about well-being-related aspects of school and learning environments from children’s perspectives that were previously so self-evident that they were rarely mentioned or so unthinkable that they were not addressed.

## Methodology

The broader research context and methodological approach of our study refer to the multinational qualitative study on Children’s Understandings of Well-being (CUWB) that investigates how children themselves conceptualize and experience well-being with an internationally comparative, context-sensitive, and cross-cultural approach (see Fattore et al., 2019, 2021). A central characteristic of the CUWB study is the understanding of well-being as a social and cultural construct, as opposed to defining well-being as an objective or subjective construct (see Fattore et al., 2019). This picks up discussions that have critically highlighted the normativity and cultural contingency of well-being in the course of an increased inclusion of positive indicators in child indicator research (Andresen, 2014; Fattore et al., 2019; Fegter, 2021). According to the discussions, not only the degree or extent of objective and subjective well-being depends on social and cultural factors but also how well-being is experienced. As Kitayama and Markus write, “it is not just that different things make people happy in different cultural contexts—this is obviously the case. More significantly it is the ways of ‘being well’ (…) that are different (2000: 114–115), depending on how the concept of ‘well’ and ‘being’ are defined and practiced.”

The aim of the CUWB study to this background is to reconstruct child well-being as a social and cultural construct from the perspective of children. The CUWB study is thus methodologically located in the sociology of childhood approach with its central concepts of the child as a social actor (Corsaro, 2017) and of childhood as a generational order (Alanen, 2009). One of the challenges for research that starts from children’s perspectives is to not authenticate children’s voices (Fattore et al., 2019; Hunner-Kreisel & Kuhn, 2010; Machold, 2015), and instead to systematically include the social and cultural contexts into the analysis. For this purpose, the CUWB study uses various epistemological approaches, e.g., standpoint theory, the sociology of knowledge or discourse theory (see Fegter, 2021), as well as ecological and socio-spatial theories. The data collections follow a shared research protocol that involves qualitative interviews, participant observations, or group discussion and applies a variety of child-oriented methods that aim to offer children appropriate forms of articulation in the context of each specific local condition (Fattore et al., 2021; Mogensen et al., 2023).

The Berlin part of the CUWB study started in 2015 and follows the principles and concepts of the CUWB protocol with changing thematic focuses (e.g., urban well-being, digital well-being, well-being during the Covid-19 pandemic, and well-being in out-of-school learning contexts), conducting qualitative interviews, city walks, and participant observations with children between the ages of 8 and 12 years old. This paper is based on semi-structured qualitative interviews that we conducted in Berlin in the spring of 2021 when Germany had been under a nationwide lockdown for several months. All children were, with only few exceptions, at home at the time experiencing distance learning via digital platforms or other digital media. The interviews were conducted as video interviews, one to one, via WebEx. We interviewed nine children between the ages of 11 and 14 who had attended a youth group with a Christian background in the eastern part of Berlin before the pandemic.

The sampling was aimed at interviewing a socially diverse group of children. Several authors such as Budde et al., (2022a, 2022b) and Andresen et al. (2020) note that children rarely spoke for themselves in studies on the pandemic and that when they did, the samples were often socially biased. By building on an existing contact to the youth group, which is attended by children from very different socioeconomic backgrounds, we were able to reach and include children in the study who most likely would not take part in others studies. Our sample thus consists of children from different socioeconomic backgrounds. However, all except one attend a gymnasium, which is a type of school with a strong emphasis on academic learning (maybe comparable to British grammar schools or US preparatory high schools).

For approaching the children and obtaining their consent, we followed an ethical approach that makes the children’s own decision crucial for their participation in the interviews and treats consent as an ongoing process (e.g., Fattore et al., 2016: 21ff.). In order to enable informed consent, the children were informed in detail and in advance about the aim of the project and the procedure and content of the interviews and the data processing. They were also informed about their right to refuse to answer, and it was made clear that participation is voluntary at any time. By choosing a way of explanation that was appropriate to the age and individual needs of the children, it was ensured that the children understood and were able to comprehend all information. While the research practice in Germany has long been dominated and shaped by the assumption that children’s participation in scientific studies legally requires parental consent, recent considerations indicate that this is not necessarily the case (Fischer & März, 2023). However, for ethical reasons and to provide reassurance to both parents and children, we decided to involve the parents in the process and obtained their consent before conducting the interviews.

The interviews were all conducted digitally via a video conferencing tool due to the pandemic restrictions. All the children were free to choose from where they did the interviews; eventually, they all took part from their children’s room, sometimes in the presence of their siblings with whom they share the room. They were also free to choose whether to leave the camera on or off. Some of the children seemed grateful for the opportunity to be allowed to turn the camera off. In the interviews, they were asked to talk about their everyday experiences during the pandemic, using the adapted CUWB protocol for the Covid-19 experiences, which are about family, leisure time and friendships, and school experiences.

For the analysis, we worked with a discourse analysis approach (Fegter, 2021). Its concept of discourse refers to a praxeological reading of Foucault (Wrana, 2015). Different to approaches that conceptualize discourses as distinct formations consisting of a number of statements, the praxeological reading defines discourse as a performative, iterative practice of relating objects, concepts, subject positions, and strategies (Foucault, 2010/1972: 46) and takes place at the micro-level of each statement. This discursive practice is conceptualized as a cultural context through which individuals’ practices of meaning-making are enabled and limited at the same time. The Berlin CUWB study adopts this approach, analyzing *what* children say and *how* discursive practices constitute their understandings of well-being as a cultural construct in situ. The analytical questions for the interview data were:

1. What advantages and disadvantages of distance learning during the school lockdowns do the interview participants highlight?
2. Which differentiations, norms, concepts, and subject positions constitute these statements?
3. Which concepts of school-related well-being emerge with the statements and which structural features of school can be reconstructed as relevant for their well-being in learning environments?

The experience of contingency is methodologically fruitful as it invites to highlight and construct similarities and differences between schooling before and during the pandemic as well as to assess and evaluate these differences (e.g., as better or worse, new or the same, important or unimportant, or normal or unusual). Analytically speaking, it prompts a way of speaking that involves *evaluative differentiations*, which help to (re)construct how concepts of well and being as well as concepts of school-related well-being are (re)produced and shifted and thus to reconstruct well-being as a cultural construct.

## Findings on Visibility as a Key Element of School-Related Well-Being from Children’s Perspective

### Less Exposure, More Joy, Being Embodied: The Learning Self in the Context of New In/Visibilities in Online Teaching

Visibility, as a key feature of schools and well-being from children’s perspective, is one of the first preliminary findings that is outlined in more detail at a micro-analytical level in the following section, starting with a statement from Anton, a 13-year-old boy, who is asked by the interviewer what he sees as an advantage of the current school situation. Anton answers:

“Um, with me it is that you can simply turn off the camera when you//you cannot do that, uh, in live classes, you cannot just put a cardboard box over yourself so that the others cannot see you. And there (.) it is just a click and (.) the camera is off. […] Or you can simply mute yourself […].”

Anton (13 y.), lines 430–435

Anton is referring to the *video conferencing* tools that mediate the online classroom interaction. Being able to mute your microphone, to turn off your camera, and thus to limit one’s own visibility and audibility, are something that he highlights as positive and as an advantage compared to the former “live classes” as he calls them. The image or allegory of putting a cardboard box over oneself gives an idea what the positive aspect is about, namely, to be able to hide oneself (“to put a cardboard box over oneself”) and to hide oneself from the gaze of others (“so that the others cannot see you”). School comes into focus here as a place that exposes the individual constantly to the gaze of others, the institution, the classmates, the teachers, and where it is difficult to find a place to hide or withdraw. Online learning is constructed as an advantage in this respect: It offers opportunities to escape, order, limit, and regulate one’s own visibility and the degree of exposure to the gaze of others in a more self-determined way, and with “just a click.”

Next to the aspect of less exposure to the gaze of others, the interviewed children address further advantages and disadvantages that they associate with the new possibilities to regulate their visibility and audibility in the digital classroom setting. Finn, a 12-year-old boy, explains:

“Well, it is that you can just go away, um, even during video conferences. No one is looking, you can just make yourself a cup of hot chocolate downstairs or get an ice cream or something.”

Finn (12 y.), lines 286–288

When asked what he likes about the current school situation, Finn mentions the possibility of briefly leaving his room during online lessons to get something to eat and drink, a hot chocolate, an ice cream (perhaps something that tastes good and that feels good). The new order of visibility and invisibility in the digital classroom allows him, from his point of view, to move more freely during class, not to be present all the time, to eat and drink something while learning, perhaps also to reward himself with an ice cream or a hot chocolate. Rosa, a 12-year-old girl, highlights something similar talking about learning from home:

“When you are at home, you can just read whenever you want and just sit down somewhere in between, just relax, and at school you can just, you just always have to keep to it.”

Rosa (12 y.), lines 53–57

This expanded scope of agency and freedom to follow individual needs and interests during class is often causally linked to non-visibility in the classroom; it is possible, as Finn says, because “no one is looking.”

Thus, visibility (the panoptic nature of the traditional school) is also constructed from the children’s perspective as a key disciplinary principle of school that subjects and disciplines their bodies, their movements, and their individual needs according to the norms of the school. This also includes the fulfilment of curricular tasks. Duc, a 14-year-old boy, tells the interviewer, for example:

“I got an exercise for my course the day before yesterday, something like ‘climbing the stairs’. Nobody does it. Nobody wants to do it. Everyone is demotivated. But at school you are just forced to do it because you are seen and stuff like that.”

Duc (14 y.), lines 462–464

Being seen is emphasized here as the critical moment that forces him and the other students to do tasks they do not really want to do.

In summary, the student’s freedom to control or regulate their own visibility and audibility in the digital classroom to a greater extent is defined here as a characteristic and an advantage of online teaching. From the children’s point of view, visibility becomes apparent as a structural principle of school that is significant to their well-being, as they first associate it with the individual’s vulnerability to the gaze of others, which they can hardly escape in the place of school, and, second, as they highlight visibility as a central disciplinary principle that subjects and controls their individual bodies, minds, and needs to the norms of the school.

The student’s needs that emerge “in the invisible,” just as the final comment, do not seem entirely inappropriate for learning processes: being able to eat and drink during learning, being able to move or get around, being able to relax in between, and being able to withdraw from others whose company could not be chosen voluntarily.

### Technologically Conditioned Agency as an Ambivalent Freedom

There is another time-related element of the new visibility order in the digital classroom, which is also associated with the phrase “it is just a click.” When asked what he sees as advantages of the current school situation, 13-year-old Anton also answers:

“Um, with me it is that you can simply turn off the camera when you//you cannot do that, uh, in live classes, you cannot just put a cardboard box over yourself so that the others cannot see you. And there it is just a click and (.) the camera is off. […] Or you can simply mute yourself […].”

Anton (13 y.), lines 430–434

The phrase “it is just a click” indicates what is not possible in the regular classroom or in the analogue world in general: to withdraw with just a small movement of the finger from the gaze of others in a matter of seconds. The changed technological conditions of classroom interaction make the regulation of one’s own visibility and audibility therefore not only possible but also extremely simple. The phrase “it is just a click” appears several times in the interviews and indicates how unusual this aspect is and how much it contrasts with the experience of school as a physical place in a geographic location that requires a much more time-consuming arrival and departure and where social expectations also make it much less feasible for individuals to simply walk away from. In the digital classroom, however, this is possible “with just a click,” and whether it was intentional, a technological problem, or an oversight usually remains unclear and is socially more difficult for others to interpret.

Considering the control and disciplinary function that the children ascribe to the structural feature of visibility in school, this means that the new technological conditions associated with the digital tools of classroom interaction shift the power balance in favor of the students: they experience a wider range of opportunity to escape from the control of the school/class/teacher at any time and “with just a click” almost effortlessly. They are in the position to decide for themselves whether and how long they show themselves and whether and how long they expose themselves, which ultimately undermine a central feature of school’s power over students. People who know that situation, e.g., with students at the university from the other perspective, probably sympathize with the teachers struggling to get the attention and to establish a symmetrical conversation in terms of visibility. Nevertheless, the children’s practices and experiences demonstrate how “powerless” the students are in the normal face-to-face lessons with respect to control over their own visibility. From this perspective, the new technological setting empowers students in their interaction with the teacher and in their relationship with the institution in the sense that they have a greater scope of action and more realistic options to decide about their presence and absence.

However, this simple and quick regulation of one’s own visibility is not only thematized as a positive freedom but also as a risky and seductive freedom as well. This becomes clear in the following passage, in which Hanna, a 14-year-old girl, answers the question of the interviewer what she does not like about the current school situation and what should change quickly from her perspective:

“And above all, you can get distracted very quickly, so if you are doing school on a laptop, then you are just one click away from Netflix or online shopping and something. That is all relatively close together. Or Instagram, that is right next to it and there is nothing to stop me from clicking on it. It is just one click. And then you get lost there and (.) it takes a while to get back.”

Hanna (14 y.), lines 322–327

Hanna problematizes that the online learning from home with her laptop quickly distracts her in class. She explains this by explaining that with the laptop one is “just one click away” from other activities such as streaming platforms or websites for online shopping. While the statement “there is nothing to stop me from clicking on it” could, isolated, give the impression that Hanna is talking about positive self-determination and freedom from external control, the following sentence, “And then you get lost there and it takes a while to get back,” links the experience with loss of control and security. There is nothing standing between Hanna and the fateful click, and if she follows this temptation, she gets lost.

In summary, the children construct the changed technological conditions of online classrooms and of regulating their own visibility and presence in the classroom as an ambivalent freedom. Although the children appreciate the new freedom and experience it as enhancing their well-being, they also deal with it in a critical way and reflect on self-responsibility to maintain control over themselves as a challenge, sometimes even as an overload.

## Conclusion

The aim of the paper was to contribute to theoretical considerations on well-being in school environments with a qualitative approach that starts from the assumption that well-being is a social and cultural construct and argues for including children’s voices into the process of knowledge production. At the level of methodology, the aim of the paper was to argue for the analytical value of the experience of contingency during the Covid-19 pandemic. As the analysis demonstrated, children’s reflections on different school routines and new learning and teaching practices provide fruitful data to reconstruct relevant aspects of school-related well-being from children’s perspectives, including aspects that might have seemed so self-evident or unquestionable before the pandemic that they were less likely to be mentioned. At the level of findings, the paper has outlined, with respect to visibility as a key feature of well-being in school environments from children’s perspectives, that they make relevant for experiences of agency, security, and self. Visibility in school is constructed as a medium of control that subjects their bodies (their need to eat, move, drink, and reward themselves while learning) to norms of the school and constantly exposes the individual self to the gaze of others leaving hardly any room for withdrawal. Conversely, visibility in the school space is also constructed as providing security, as unsupervised and unobserved actions on the internet and the digital sphere are associated with a sense of getting lost and losing control. By addressing and negotiating the relevance of visibility, the children take up a specific characteristic of school that was elaborated and became the subject of pedagogical reflections particularly through Foucault’s work on the transformation of power relations (Foucault, 1977/1975; Grabau & Rieger-Ladich, 2014; Pongratz, 2004). This shows how the experience of contingency due to radical changes caused by the pandemic and the realization that school could be different not only reveals the panoptic structure of the traditional school as a regime of visibility, it also emphasizes its relation to school-related well-being and marks this relationship (and the disciplining function of the panoptic structure) as one that becomes significant for well-being in different ways as it constrains agency on the one hand but also provides a sense of security on the other hand. In this sense, the children’s reflections inform discussions on educational well-being as they stimulate reconsiderations of the panoptic structure of school as relevant to school-related well-being from children’s perspective.

The relevance of these findings for research, practitioners, and policy arises from challenges that schools are facing. Schools have changed during the pandemic because students and teachers have changed. These current transformations need to be included into theoretical and professional concepts on school-related well-being. While the current discourse focuses on negative aspects of the pandemic for children, experiences during the pandemic with new learning arrangements also have epistemic potential. Listening to children’s voices, e.g., in regard to aspects of visibility in digital learning arrangements, provides an opportunity to re-think and improve schools systematically under the perspectives of well-being and of developing as an individual and a citizen. This does not mean to reduce well-being in schools to the idea of just happiness. A school is a normalizing institution, and the children reflect this very clearly. One conclusion would be to engage in a concept of well-being that includes the ability to place oneself in a differentiated and critical relationship to the demands of the institution and to integrate visibility as a key structural element of schools into these considerations.
